# Identification of a New Epitope in uPAR as a Target for the Cancer Therapeutic Monoclonal Antibody ATN-658, a Structural Homolog of the uPAR Binding Integrin CD11b (αM)

**DOI:** 10.1371/journal.pone.0085349

**Published:** 2014-01-21

**Authors:** Xiang Xu, Yuan Cai, Ying Wei, Fernando Donate, Jose Juarez, Graham Parry, Liqing Chen, Edward J. Meehan, Richard W. Ahn, Andrey Ugolkov, Oleksii Dubrovskyi, Thomas V. O'Halloran, Mingdong Huang, Andrew P. Mazar

**Affiliations:** 1 Division of Hemostasis and Thrombosis, Beth Israel Deaconess Medical Center, Harvard Medical School, Boston, Massachusetts, United States of America; 2 State Key Laboratory of Structural Chemistry, Fujian Institute of Research on the Structure of Matter, Chinese Academy of Sciences, Fuzhou, Fujian, China; 3 Department of Medicine, University of California San Francisco, San Francisco, California, United States of America; 4 Agensys, St. Santa Monica, California, United States of America; 5 GNF, San Diego, California, United States of America; 6 Attenuon, San Diego, California, United States of America; 7 Department of Chemistry, University of Alabama in Huntsville, Huntsville, Alabama, United States of America; 8 Chemistry of Life Processes Institute, Northwestern University, Evanston, Illinois, United States of America; 9 Robert H. Lurie Comprehensive Cancer Center, Northwestern University, Evanston, Illinois, United States of America; 10 Department of Molecular Biosciences, Northwestern University, Evanston, Illinois, United States of America; 11 Department of Chemistry, Northwestern University, Evanston, Illinois, United States of America; European Institute of Oncology, Italy

## Abstract

The urokinase plasminogen activator receptor (uPAR) plays a role in tumor progression and has been proposed as a target for the treatment of cancer. We recently described the development of a novel humanized monoclonal antibody that targets uPAR and has anti-tumor activity in multiple xenograft animal tumor models. This antibody, ATN-658, does not inhibit ligand binding (i.e. uPA and vitronectin) to uPAR and its mechanism of action remains unclear. As a first step in understanding the anti-tumor activity of ATN-658, we set out to identify the epitope on uPAR to which ATN-658 binds. Guided by comparisons between primate and human uPAR, epitope mapping studies were performed using several orthogonal techniques. Systematic site directed and alanine scanning mutagenesis identified the region of aa 268–275 of uPAR as the epitope for ATN-658. No known function has previously been attributed to this epitope Structural insights into epitope recognition were obtained from structural studies of the Fab fragment of ATN-658 bound to uPAR. The structure shows that the ATN-658 binds to the DIII domain of uPAR, close to the C-terminus of the receptor, corroborating the epitope mapping results. Intriguingly, when bound to uPAR, the complementarity determining region (CDR) regions of ATN-658 closely mimic the binding regions of the integrin CD11b (αM), a previously identified uPAR ligand thought to be involved in leukocyte rolling, migration and complement fixation with no known role in tumor progression of solid tumors. These studies reveal a new functional epitope on uPAR involved in tumor progression and demonstrate a previously unrecognized strategy for the therapeutic targeting of uPAR.

## Introduction

Metastasis and angiogenesis share many common phenotypic features that lead to the invasion and migration of tumor and endothelial cells. These include the up-regulation of protease and integrin expression, the loss of cell-cell and cell-matrix contacts, an increase in responsiveness to growth and differentiation factors, and the remodeling of extracellular matrix (ECM) and basement membrane (BM) [1], [2]. The urokinase plasminogen activator (uPA) system, comprised of uPA, a specific cell surface receptor for uPA (uPAR), and serpin inhibitors of uPA such as plasminogen activator inhibitor-1 (PAI-1), plays a central role in many of these activities [3]–[6]. The activity of this system is responsible for initiating cascades that result in the activation of plasminogen and several pro-metalloproteases (proMMPs) [7], [8], release and processing of latent growth factors deposited in the ECM such as FGF-2, VEGF, HGF, and TGF-β [9]–[12] and remodeling components of the ECM such as vitronectin and fibronectin [13], [14]. These activities are generally mediated by the proteolytic function of uPA when bound to uPAR, can be modulated by the inhibition of uPA by PAI-1, and occur in the extracellular environment. In addition, uPAR also interacts with many other ligands in addition to uPA including several integrins such as α5β_1_, α3β_1_, and α5β_3_ [15]–[17], as well as other cell surface and ECM ligands including vitronectin and G protein–coupled receptors [6]. Several of these interactions have been demonstrated to be important for tumor cell survival, invasion, and angiogenesis [6], and involve uPAR-dependent signaling.

For these reasons, uPAR has been proposed as a therapeutic target for the treatment of cancer. However, despite an abundance of literature demonstrating the importance of uPAR in the progression of most solid cancers, including breast [18], colon [19], prostate [20], pancreatic [21], ovarian [22], lung [23], and brain [24] as well as several hematologic malignancies such as acute leukemia and myeloma [25], no uPAR targeted therapeutic agent has been developed or evaluated in cancer clinical trials to date. A number of antibodies that directly inhibit the binding of uPA to uPAR have been proposed and tested in pre-clinical studies but most of these have only demonstrated modest antitumor activity and were therefore never advanced into the clinic.

Recently, we identified and developed a novel uPAR targeted monoclonal antibody that demonstrates robust antitumor effects in a number of different animal tumor models but does not block the binding of uPA to uPAR [22], [26]–[28]. This antibody, ATN-658, has several unique attributes that differentiate it from previous uPAR targeted approaches. A key feature is that ATN-658 is that it does not block uPA binding to uPAR and is able to bind to uPAR even when it is occupied by uPA, but nevertheless inhibits migration and invasion *in vitro* [22], [27]. ATN-658 has no effect on uPA mediated plasminogen activation but does have a number of effects on signaling pathways and the expression of various genes implicated in tumor progression when evaluated in models of prostate and ovarian cancer *in vitro* and *in vivo* [22], [27]. One of the most striking observations with ATN-658 is that the pharmacological targeting of uPAR by this antibody leads to robust antitumor effects in a broad range of solid tumor xenograft models [22], [26]–[28]. Antitumor effects have been observed regardless of tumor histology in these models and in addition to inhibition of metastasis *in vivo*, as would be predicted for an uPAR targeted agent, ATN-658 is also able to inhibit tumor proliferation and induce apoptosis [22], [26], [28]. However, the structural and mechanistic basis for these antitumor effects remain unclear.

In order to address this issue, we characterized the epitope on uPAR to which ATN-658 binds. The epitope for ATN-658 was initially mapped using site-directed mutagenesis and confirmed by a novel deuterium exchange mass spectrometry technique (H/D-exchange mass spectrometry, ExSAR). This epitope was determined to be an epitope for which no function in uPAR has previously been described. In addition, we determined the crystal structures of the ATN-658 Fab fragment alone and in complex with uPAR, the amino terminal (uPAR binding domain) of uPA (ATF), and the somatomedin B domain (SMB) of vitronectin. The ATN-658 Fab binds to the DIII of uPAR, consistent with the epitope mapping results. The epitope on uPAR for ATN-658 is close to the C-terminus, and has no overlap with the urokinase and vitronectin binding sites. This study also revealed structural homology between the ATN-658 CDR loops and the uPAR binding region of integrin αM. Moreover, we showed that ATN-658 binding blocks the association of another integrin, α5β1, to uPAR and thus impairs integrin α5β1-mediated adhesion to extracellular matrix. These results suggest a previously unrecognized mechanism by which uPAR may function in tumor progression and a novel epitope for the therapeutic targeting of this receptor.

## Materials and Methods

### Cell Lines

Human tumor lines PC-3 (prostate adenocarcinoma), HeLa (cervical carcinoma), and human lung cancer cell line H1299 were obtained from the American Type Culture Collection (Manassas, VA). Both cell lines were cultured in Dulbecco's modified minimum Eagle's medium supplemented with 10% fetal bovine serum and penicillin-streptomycin. Mouse tumor cell line B-16 (melanoma), dog osteosarcoma (D-17) and immortalized African green monkey (AGM) kidney cells (COS-1) were verified to express uPAR by RT-PCR and western blot using a rabbit polyclonal uPAR antiserum (rD2D3) known to cross-react with uPAR from multiple species as previously described [29]. uPAR expressing cells were then used to evaluate the ability of ATN-658 to cross-react with various non-human uPAR. Both recombinant soluble uPAR (suPAR, residues 1–277) and ATF (amino acid residues 1–143 of uPA) were produced in Drosophila S2 cells as secreted proteins *E.Coli* [31].

### Characterization of monoclonal antibody ATN-658

ATN-658 was raised against a chymotryptic fragment of soluble uPAR (suPAR) comprising DIIDIII (aa 88–283 of mature uPAR) expressed in *Drosophila* S2 cells, using standard techniques. Briefly, Balb/c mice were immunized with suPARDIIDIII fragments conjugated to KLH and the magnitude of the immune response monitored by ELISA. Based on these data, hybridomas were generated by fusing spleen cells with the myeloma cell line P3x63Ag8.653. Frozen stocks of 10 parental hybridomas were made and five and were purified as described [30]. The SMB domain protein (amino acid residues 1–50 of human vitronectin) was a kind gift of Dr. Aiwu Zhou, expressed in of the hybridomas subjected to limiting dilution. Tissue culture supernatants from these monoclonal antibodies were then assayed for activity in ELISA assays and the isotype of each antibody determined using IsoStrips (Roche).ATN-658, isotype IgG1κ, bound suPAR immobilized to plastic with a K_D_ of ∼1 nM and iodinated ATN-658 specifically bound uPAR on the surface of HeLa cells with a K_D_ of ∼5 nM. The Kd of ATN-658 for suPAR was also confirmed using surface plasmon resonance (BIAcore). Western blot analysis demonstrated that ATN-658 was specific for human uPAR and did not cross-react with mouse uPAR. ATN-658 was purified from tissue culture supernatant by column chromatography using protein-A Sepharose, typical yields ranged from 60–120 mg/L of tissue culture supernatant and the purity of the final material was >95% as determined by HPLC. Biotin-ATN-658 was prepared for whole cell binding assays and for flow cytometry as previously described [27].

### Preparation of ATN-658 Fab fragments

ATN-658 Fab fragments were prepared by extensive proteolytic digestion of the purified antibody (5 h, 37°C) with immobilized Papain (Pierce). ATN-658 Fab fragments were separated from the antibody Fc domains by protein-A affinity chromatography and the purified Fab fragments characterized by SDS-PAGE. To confirm that the purified ATN-658 Fab fragments retained the ability to bind uPAR with high affinity we performed competition assays using biotinylated ATN-658 [27] and immobilized suPAR. The protein was concentrated to 5 mg/ml using Millipore Ultrafree centrifugal filters for protein crystallization.

### Evaluation of binding of ATN-658 to cells in vitro

The species specificity of ATN-658 binding was evaluated by whole cell binding assays or flow cytometry as previously described [27]. Whole cell binding assays were performed on cells (300,000/well) plated on gelatin coated 12-well plates (Costar #3516) and allowed to attach overnight. After extensive washing with Hank's buffered salt solution (HBSS; Invitrogen, Inc.) containing 0.1% bovine serum albumin (BSA; Sigma) adherent cells were incubated with varying concentrations of biotin-labeled ATN-658 in HBSS/0.1% BSA for 1 h at room temperature. After washing 3-times with HBSS/0.1% BSA cells were incubated with HRP-conjugated streptavidin for a further 0.5 h at RT, wells washed as described above and bound antibody detected by incubation with OPD substrate (Sigma). After color development 0.1 mL of substrate was transferred from each well to a 96 well plate, the reaction stopped by the addition of 20 µL 1MH_2_SO_4_ and the absorbance at OD-490 nm recorded. Prior to flow cytometry adherent cells were harvested with trypsin and resuspended in FACS buffer (2% fetal bovine serum in PBS). Cells (2×10^6^ cells in 200 µL FACS buffer) were incubated with ATN-658 (final concentration 10 µg/mL), control mouse IgG, a rabbit polyclonal antibody (1∶100 dilution) raised against a fragment of human uPAR (rDIIDIII) or normal rabbit serum (NRS) for 1 h at 4°C. Following incubation, cells were washed three times with 1 mL FACS buffer and resuspended in 100 µL of FACS buffer containing either goat anti-rabbit or goat-anti mouse Alexa Fluor 488 conjugated secondary antibodies (Invitrogen, Inc.) and incubated for 30 minutes at 4°C. Cells were washed as described above and resuspended in 0.5 mL FACS buffer prior to acquisition of flow cytometry data.

### Site-directed mutagenesis studies of ATN-658 binding to primate uPAR

African green monkey (AGM) suPAR was cloned from COS-1 cells by PCR, sequenced and the protein expressed as previously described for human suPAR [30], [32]. Site-directed mutagenesis of the AGM suPAR plasmid template was carried out using a Quikchange site-directed mutagenesis kit (Stratagene, Inc.), according to the manufacturer's instructions, to generate nine mutants in which each amino acid unique to AGM suPAR was replaced by the corresponding human suPAR sequence. Sequencing and restriction digestion of the each mutated plasmid was used to confirm the sequence change and that the remaining sequence remained intact. AGM mutant suPAR cDNA was then subcloned into a Drosophila expression vector (pMT/BiP/V5-HisA) containing the V5 epitope flag and used to transfect S2 cells. Small scale (500 mL) shaker cultures were established to produce protein for experiments and protein expression was confirmed by western blot using a pAb [29] against uPAR that cross-reacted with primate suPAR. Culture supernatants (CS) for each clone were clarified by centrifugation and aliquots immune-precipitated using ATN-658 and protein-G Sepharose. Bound proteins were detected by western blot using the cross-reacting rabbit anti-DIIDIII polyclonal antibody described above. Another uPAR MAb, ATN-615, which is also species specific for human uPAR and for which the epitope was already known from x-ray crystallography studies [33], [34], was used as a control to validate this IP method.

Alternatively, culture supernatants were analyzed by capture ELISA using an anti-V5 antibody and biotinylated anti-uPAR antibodies ATN-658 and ATN-617. ATN-617 [27] is a monoclonal antibody that binds to a different epitope on uPAR and cross-reacts with AGM suPAR. Plates were coated with V5 antibody (1 mg/mL) in PBS (100 µL well in a 96 well EIA/RIA high binding plate). After 3 h incubation at RT, wells were washed and then incubated with 1× casein/water (200 µL/well) for 2 h at RT to block non-specific binding. Wells were washed and 100 µL of culture supernatant was added to each well and incubated O/N at 4°C. Culture supernatants were estimated to contain ∼3 ug/mL of suPAR. Biotin ATN-658 or biotin-ATN-617 (1 µg/mL) was then added to the wells and incubated for a further 0.5 h at room temperature followed by extensive washing. Finally, Streptavidin-HRP was added and after additional incubation and washing, color was developed using OPD and bound ATN-658 or bound ATN-617 was detected by absorbance measured at 490 nm, as previously described [27].

### Epitope mapping of ATN-658 by H/D-exchange mass spectrometry (H/D-Ex)

1.5 mg of ATN-658 Fab fragment was immobilized onto 200 mg POROS AL resin (Applied Biosystems) according to the manufacturer's instructions. Pull-down experiments were performed using suPAR-DIIDIII to confirm the specificity and binding capacity of the affinity column. ATN-658 Fab conjugated beads (353 µL) were resuspended in 700 µL of deuterated phosphate buffer (50 mM KH_2_PO_4_, 50 mM NaCl, pH 7.4) and allowed to equilibrate at 4°C.suPAR-DIIDIII was resuspended in 40 µL D2O pH 7.4 (Cambridge Isotopes) for either 150, 500, 1500 or 5000 seconds to allow complete deuteration of surface amides. Labeled suPAR-DIIDIII and affinity resin were then mixed together for 10 min at 4°C. Back exchange of solvent exposed amides was carried out by replacing the ^2^H phosphate buffer with H_2_O and incubating at 4°C for a time equal to the labeling step. A control experiment was carried out by binding unlabeled suPAR-DIIDIII to ATN-658 Fab conjugated beads and labeling the bound material by incubation with 700 µL of deuterated phosphate buffer for the times listed above. Back exchange was then performed as described. The reactions were quenched and suPAR-DIIDIII eluted from the affinity column using 40 µL 0.8% formic acid. Following the addition of 20 µL 8 M urea, 1 M TCEP, pH 3.0 the eluted material was injected into the Hydrogen/Deuterium Exchange Mass Spectrometry (H/D-Ex) platform (ExSAR, Monmouth Junction, NJ) consisting of tandem immobilized pepsin and C18 separation columns. The digested fragments were separated and analyzed by mass spectrometry and the identities of each peak identified by comparison with data obtained in control experiments using deuterium-labeled and unlabeled suPAR-DIIDIII digested with pepsin.

### Protein crystallization and X-ray data collection

The suPAR-ATF complex was formed by incubating ATF with suPAR at room temperature in 50 mM HEPES and 100 mM NaCl pH 7.4 and was purified on a Superdex 75 gel filtration column. The eluted complex was then added excess ATN-658 Fab, and the ternary complex ATN-658-uPAR-ATF was purified again on a Superdex 200 gel filtration column. Prior to the crystallization, SMB was added to the ternary complex at a 2∶1 molar ratio, and then concentrated to 10 mg/mL without any further purification. Crystallization was carried out using the sitting drop vapor diffusion method at 22°C. The diffracting quality ATN-658 Fab crystals were generated using 20–22% PEG 3350, 0.1 M Tris pH 8.0–8.5. For crystallization of the ATN-658-uPAR-ATF-SMB quaternary complex, the protein at 10 mg/mL was mixed with an equal volume of reservoir solution containing 0.1 M HEPES pH 7.5, 55% (v/v) Tacsimate, and 2% (v/v) 2-methyl-1,3-propanediol. Triangle crystals were typically appeared after a couple months and grew to a final size of 0.15×0.15×0.15 mm^3^.

The ATN-658 Fab crystals were cryo-protected with the addition of 20% glycerol to the mother liquor. Diffraction data was collected at 100°K on the Advanced Photon Sources (beamline 22-ID, SER-CAT).X-ray diffraction data collection for the complex was carried out at 100 K on the NSLS Brookhaven National Laboratory (beam line ×29). Most ATN-658-uPAR-ATF-SMB quaternary crystals diffracted very weakly, usually less than 6 Å. This is presumably due to high solvent content (76% solvent) and the presence of a long axis of the crystals (c = 391.58 Å). After numerous trials with different crystals, different data collection strategies and the exploration of different cryo-protectant solutions. Diffraction data was collected from a flash-cooled crystal which was cryo-protected by a sequential soaking into mother liquid with increased concentration of ethylene glycol to the final concentration of 10% (v/v) to 4.6 Å resolution. The diffraction data were indexed and processed using the HKL2000 program [35]. The data collection and final structural refinement statistics for both structures are integrated in Table 1.

**Table 1:** Statistics of X-ray data collection and structural model refinement.

Crystals	ATN 658 Fab	ATN-658-uPAR-ATF-SMB
**X-ray source**	APS SER-CAT beamline 22-ID	**BNL ×29**
**Temperature (K)**	100	**100**
**Resolution range**	31.45-1.60 (1.64-1.60)^a^	**26.90- 4.50 (4.66-4.50)** a
**Wavelength (Å)**	1.04	**1.04**
**Space group**	P12_1_1	**H32**
**Cell parameters (Å)**	37.31, 132.30, 46.83	**164.65, 164.659, 391.582**
**Unique reflections**	49312 (4021)	**12451 (1221)**
**Rmerge (%)** b	0.094 (0.290)	**0.139 (0.841)**
**Completeness (%)**	85.7(70.0)	**99.7(100.0)**
**Average I/σ**	9.2 (4.65)	**16.14 (2.03)**
**Data redundancy**	3.0 (2.6)	**4.4 (4.5)**
**Model refinement:**		
**R-factor/Rfree (%)**	19.8/24.6	**20.7/29.3**
**Overall B-factors (Å^2^)**	23.0	**61.436**
**Protein B-factors (Å^2^)**	21.3	**259.694**
**Solvent B-factors (Å^2^)**	39.4	
**R.m.s.d. from ideal bond length (Å)**	0.012	**0.013**
**R.m.s.d from ideal bond angles (°)**	1.407	**2.018**
**Ramachandran plot, % residues in regions:**		
**favored**	93.8	**84.5**
**allowed**	5.7	**13.7**
**outlier**	0.5	**1.8**
**PDB ID**		

a Numbers in the parentheses are for the highest resolution shells;

b R_merge_ = ΣhΣi|Ii(h)-<I(h)>|/ΣhΣiIi(h), where <I(h)> is the mean intensity of reflection h.

### Structure determination and refinement

The structures of the ATN-658 Fab was solved by molecular replacement method (MOLREP [36]of the CCP4 program suite [37]) using an antibody structure (PDB entry 2DDQ) as a search model. The model was then subjected to several rounds of manual building using Coot [38] alternated with restrained refinement using Refmac5 [39]. In the final cycles of refinement, TLS with twenty TLS groups for each chain (generated by the TLS motion determination (TLSMD) server [40] was included in the refinement.

This ATN-658 Fab structure was then used as a molecular replacement model to solve the structure of the quaternary complex (ATN-658-uPAR-ATF-SMB) by molecular replacement method using programs MOLREP [36] and CNS [41]. A suPAR-ATF model (PDB entry 1fd6 [33]) was then positioned into the crystals of the quaternary complexes by molecular replacement (molrep [36]). Despite the low resolution of this crystal (4.5 Å), very strong molecular replacement solutions for both models were obtained. After refinement using the CNS program [41], the Fo-Fc difference electron density showed the electron density for SMB domain of vitronectin, further confirming the correct molecular replacement solutions. The resulting models with all four protein components were further refined using CNS v1.3 [41]and REFMAC [39], and manually adjusted by program O [42] or Coot [38].

All final structures were analyzed and validated by PROCHECK [43], PYMOL [44] and MOLSOFT ICM [45]. The coordinates of the reported structure have been deposited in the Protein Data Bank (PDB). The final statistics, validation, and stereochemical quality for the structure are reported in Table 1.

### Co-immunoprecipitation

HT1099 cells were lysed in Triton lysis buffer (50 mMHepes, pH 7.5, 150 mM NaCl, and 1% Triton X-100) supplemented with protease inhibitors and 1 mM PMSF. Clarified lysates were immunoprecipitated with antibodies ATN-615 and ATN-658. The immunoprecipitates were blotted for uPAR (R2) or integrin α5 (pAb).

### Cell adhesion assay

The cell adhesion assay was performed as described previously [46]. In brief, H1299 cells were incubated in DME/0.1% BSA with 500 µM RGD or RAD peptides for 1 h at 37°C and were then seeded onto fibronectin (5 µg/ml, Sigma)–coated plates with or without ATN-615. After washing, attached cells were fixed and stained with Giemsa. The data were quantified by measuring absorbance at 550 nm.

## Results

### ATN-658 does not bind to non-human uPAR

**Figure 1:**
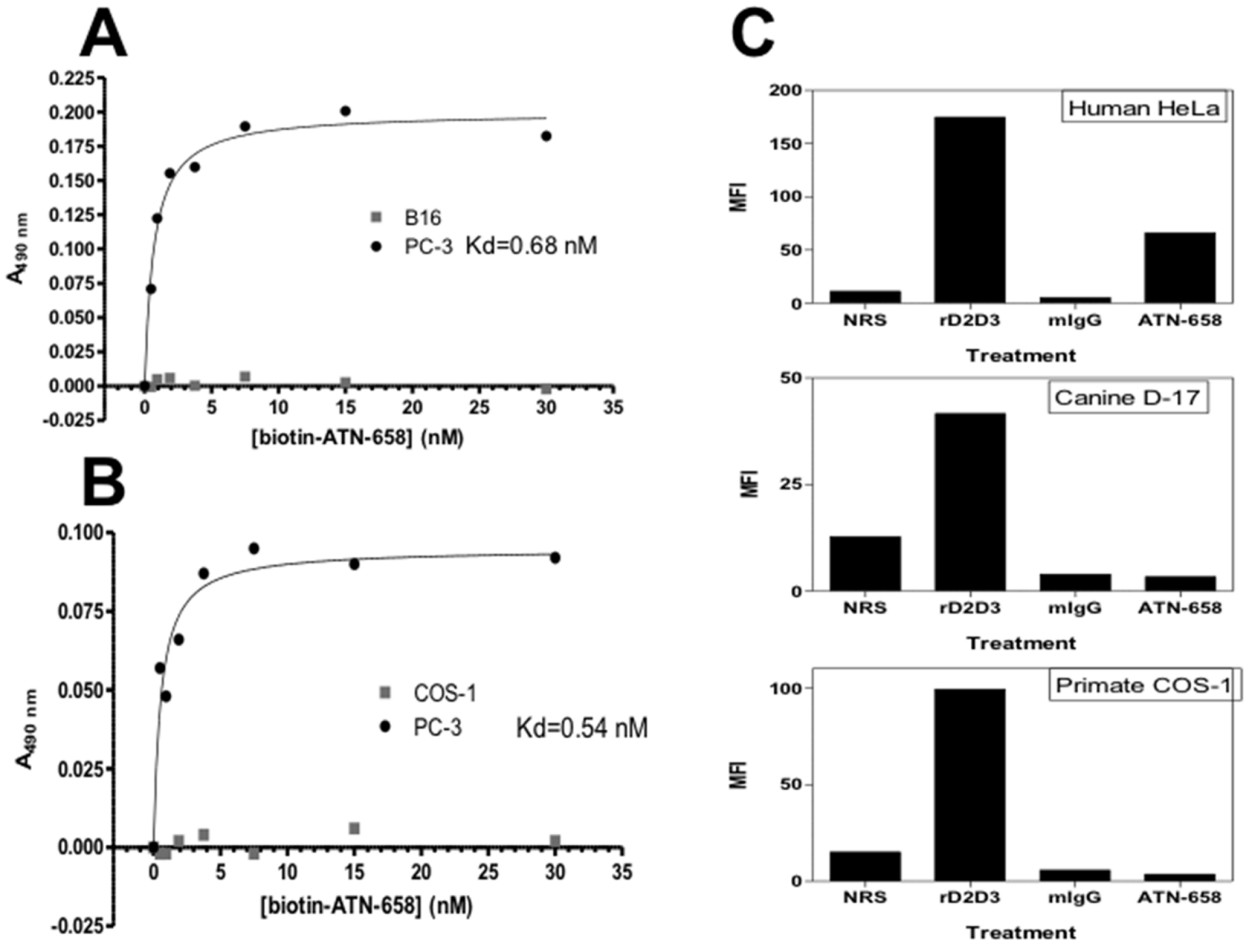
Cross reactivity of ATN-658 with uPAR from various species. A & B. The specificity of ATN-658 was measured by direct binding assays using uPAR expressing mouse melanoma (B16), human prostate carcinoma (PC-3) and African green monkey (COS-1) cells and biotin-labeled ATN-658. B. FACS analysis was performed using HeLa cells (expressing human uPAR), D-17 lung carcinoma cells (expressing canine uPAR and COS-1 cells. Each cell line was incubated with normal rabbit serum (NRS), a rabbit polyclonal antibody raised against a fragment of human uPAR (rDIIDIII), mouse IgG (mIgG) or ATN-658 and the appropriate FITC labeled secondary antibody.

Initial studies evaluated the binding of ATN-658 to cells from various species that expressed uPAR. uPAR expression was confirmed in all cell lines initially by RT-PCR followed by western blot using a rabbit anti-human uPAR pAb (rD2D3) that cross-reacts with uPAR from rodent and dog. This study was undertaken to identify possible animal species that could be used for future toxicology studies of ATN-658. Biotin-ATN-658, which retained the full binding activity of unmodified ATN-658 [27], was used for this evaluation. Biotin-ATN-658 did not bind to mouse melanoma (B16) cells (Fig. 1A) or African green monkey (AGM) (Fig. 1B) immortalized kidney cells (COS-1) in whole cell saturation binding experiments, whereas saturable binding was observed to the uPAR expressing human prostate cancer cell line, PC-3 (Fig. 1A&B), as previously described [27]. These results were confirmed and extended to include dog osteosarcoma cells (D-17) using non-biotinylated ATN-658 and an isotope matched IgG as a negative control and evaluated by flow cytometry (Fig. 1C).

### Identification of the epitope for ATN-658 on human suPAR by site-directed mutagenesis

**Figure 2:**
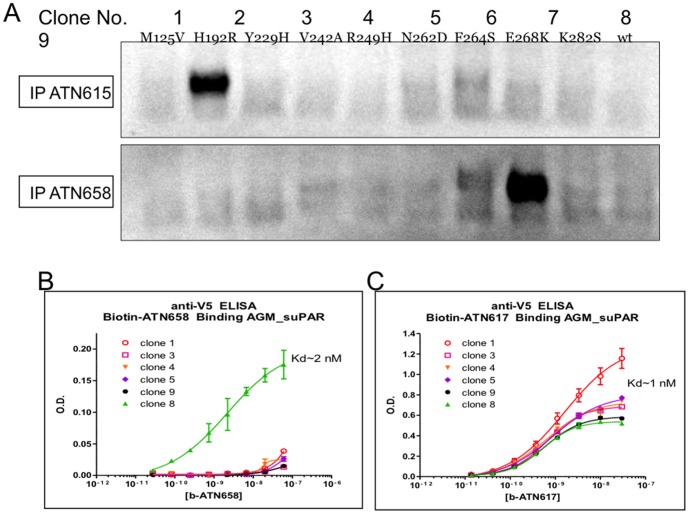
Immunoprecipitation of AGM suPAR clones using ATN-615 and ATN-658. **A.** Single point mutants were introduced into AGM suPAR such that a single amino acid was changed from the AGM sequence to the human uPAR sequence and the proteins expressed and purified as described in the Materials and Methods. Each clone is identified by number (1–9) with the point mutation identified below the number using the single letter amino acid code. Except for the single point mutation, the rest of the suPAR sequence is that of AGM. suPAR mutants were expressed in S2 cells and supernatants incubated with either ATN-615 or ATN-658 followed by immunoprecipitation as described in Materials and Methods. suPAR was then detected by western blot using a rabbit polyclonal antiserum raised against human suPAR. **B.** Capture ELISA assays were used to measure the binding affinity of ATN-658 for the various suPAR clones described in (A). Capture on the ELISA plate was through the V5 tag and detection used biotin-ATN-658. **C.** Biotin-ATN-617 was used in a similar ELISA format as described in (B) to confirm that introduction of the single point mutation did not have a global effect on suPAR conformation.ATN-617 cross-reacts with monkey suPAR and all clones appeared to retain this reactivity after introduction of the mutation.

Sequence alignment of primate DIIDIII uPAR sequences revealed that human and AGM uPAR only differed at nine amino acid positions (Table 2). These nine amino acid differences were sufficient to completely abrogate the binding of ATN-658 to AGM uPAR. Thus, we cloned and expressed AGM suPAR in S2 cells and made nine different mutants that sequentially replaced one amino acid from the AGM sequence in each mutant with the corresponding human amino acid (Fig. 2A). The initial evaluation of these mutants was qualitative where the ability of ATN-658 to immunoprecipitate (IP) a particular mutant from the culture supernatant (CS) from cells expressing that mutant was assessed. ATN-615, which is also human uPAR specific but for which we had already identified the epitope [33], [34], was used to confirm the utility of this approach. ATN-615 was only able to IP the H192R suPAR mutant (clone 2; Fig. 2A). This is consistent with the described epitope for ATN-615, which is comprised of aa 187–192 (Fig. 2A). In fact, the only amino acid difference between AGM and human uPAR in this epitope is at aa 192. Similarly, ATN-658 was only able to IP the E268K (clone 8) suPAR mutant implicating this residue as part of the ATN-658 epitope. Alanine scanning mutagenesis around this amino acid maps out the sequence from aa 268–275 as the epitope for ATN-658 (S1). Since IP is a qualitative assessment of binding, capture ELISA assays were set up to measure the actual affinity of ATN-658 for the various AGM suPAR mutants as it was possible that perhaps more than aa 268 was required in order to restore full binding activity for ATN-658 when the AGM suPAR sequence was mutated to the human sequence. Since the AGM suPAR mutants were expressed with an incorporated V5 flag, an anti-V5 flag antibody was used to capture AGM suPAR on solid phase. The binding affinity could then be determined for each AGM mutant using saturation binding studies as previously described for ATN-658 [27]. Using this approach, the binding of ATN-658 was only observed to clone 8 with a K*_d_*∼2 nM, similar to the K*_d_* of ATN-658 for human suPAR and for human uPAR expressing cells indicating that this single amino acid change was responsible for complete abrogation of ATN-658 binding to AGM suPAR (Fig. 2B). This single amino acid change did not abrogate ATN-658 binding through a global effect on suPAR conformation since the binding of ATN-617, which binds to DIIDIII and does cross-react with AGM suPAR, was not altered (Fig. 2C).

**Table 2:** Amino acid sequence differences in DIIDIII of uPAR from human, African green monkey (AGM) and cynomolgus monkey (crab eating macaque).

suPAR Amino Acid #	Human	*Cercopithecus aethiops* (African Green monkey COS-1)	*Macaca fascicularis* (Crab eating or Long tailed Macque)
125	V	M	V
152	G	G	S
157	N	N	S
192	R	H	H
229	H	Y	Y
242	A	V	V
249	H	R	R
259	N	N	H
262	D	N	N
264	S	F	S
268	K	E	E
278	V	V	I
282	S	K	K

Alignment of this epitope across multiple species of uPAR helps explain the species specificity of ATN-658 binding (3). The old world primates (apes, chimpanzees) are evolutionarily nearer to man and have homologous uPAR to human in the epitope for ATN-658. ATN-658 binding has been observed by immunohistochemistry to chimpanzee tissues consistent with this observation. In contrast, new world primate (macaques, AGM) uPAR differs in most cases at a single residue, aa 268, and this is sufficient to completely abrogate the binding of ATN-658. The uPAR of lower order mammals also differs at this position as well as others consistent with the lack of binding of ATN-658 to uPAR or cells expressing uPAR from these species as well (Table 3).

**Table 3:** Sequence alignment of the ATN-658 epitope in primates and lower order mammals.

	268 277
Human	KSGCNHPDLD
Bonobo	KSGCNHPDLD
Chimpanzee	KSGCNHPDLD
Gorilla	KSGCNHPDLD
Orangutan	KSGCNHPDLD
Pigtailed Macaque	***E***SGCNHPDLD
Rhesus Macaque	***E***SGCNHPDLD
Long-tailed Macaque	***E***SGCNHPDLD
African Green Monkey	***E***SGCNHPDLD
Ring-tailed LEmur	***G***SGCNHP***AR***D
Northern Owl Monkey	***END***CN***N***P***AE***D
Dog	T***GNS***CNHP***I***LD
Mouse	***HG***SGCN***S***P***TGG***
Rat	***NG***SGCN***R***P***TGG***
Hamster	***DGD***GCN***G***P***RSG***

A single amino acid change in this epitope is sufficient to confer species specificity of ATN-658 for human uPAR.

### Confirmation of ATN-658 epitope by Hydrogen/Deuterium Exchange Mass Spectrometry (H/D-Ex)

**Figure 3:**
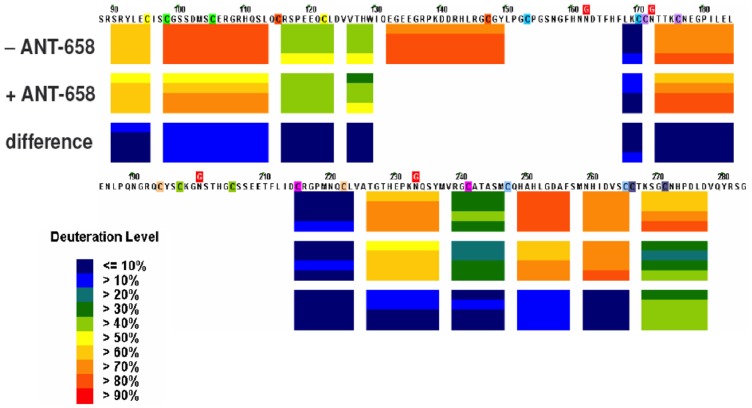
EXSAR difference map between ATN-658 bound and free suPARDIIDIII. EXSAR analysis was carried out as described in Material and Methods.

Hydrogen-deuterium (^1^H-D) exchange is a useful tool for identifying protein binding sites or interfaces. Following transfer from water to a deuterium-based solvent system (heavy water) a protein will experience an increase in mass as the protein hydrogen atoms are gradually replaced with deuterons. The likelihood of a hydrogen-deuterium exchange event is largely determined by protein structure and solvent accessibility. Differences in the rate of amide hydrogen exchange identify the location of an epitope. When an antibody binds to a protein target, surface regions that exclude solvent upon complex formation exchange much more slowly. Thus, solvent excluded regions are useful for deducing the location of a binding site. Using this approach, ^1^H-D exchange was measured for DIIDIII suPAR with and without ATN-658 bound as described in Methods and a difference map determined (Fig. 3). Differences in ^1^H-D exchange in blue demonstrated no difference between bound and unbound DIIDIII suPAR whereas differences in green suggested at least a 40% difference in exchange and identified the epitope of ATN-658 on suPAR. The only sequence where this difference was observed in the presence of ATN-658 was between aa 268–277 (Fig. 3), confirming the epitope identified using site directed mutagenesis.

### Crystal structures of the ATN-658 Fab and its complex with uPAR, ATF and SMB reveal the binding epitope of ATN-658

To further evaluate the binding epitope of ATN-658, we determined the crystal structure of ATN-658 and its complex with human soluble uPAR. We crystallized the quaternary complex of ATN-658, suPAR, ATF, and somatomedin B domain (SMB) of vitronectin. The presence of the SMB was absolutely necessary to obtain the protein crystals. The crystals diffracted to only 4.5 Å, despite numerous optimization efforts on protein crystallization and X-ray data collection strategies. This is presumably due to high solvent content (76% solvent) and the presence of a long axis in the crystals (c = 391.58 Å).In order to solve this low resolution structure using molecular replacement, we also crystallized the free Fab fragment of ATN-658, and determined its structure to a high resolution (1.6 Å, Table 1). This structure of ATN-658 Fab fragment, together with the structures of uPAR-ATF-SMB [47], allowed us to unambiguously solve the quaternary structure of ATN-658-uPAR-ATF-SMB using molecular replacement methods (Table 1).

The 1.6 Å crystal structure of the ATN-658 Fab alone consists of residues 1–127 and 134–212 of the heavy chain and residues 1–213 of the light chain. The antigen-binding site located at the end of the variable domains, comprised of six CDRs, is clearly defined in this antigen-free structure. The H3 loop lies in the center of the pocket surrounded by the light-chain CDRs L1, L2, and L3 and the heavy-chain CDRs H1 and H2, with both hydrophobic and hydrophilic interactions with the other CDRs. The six CDRs form a relatively flat but undulating surface, a feature of anti-protein antibodies. In addition, the conformations of the 6 CDRs in the free form are quite similar to the conformations in the antigen-bound form (RMSD of 0.49 Å for Cα atoms in 427 residues). These results indicate that ATN-658 has structurally rigid CDRs.

**Figure 4:**
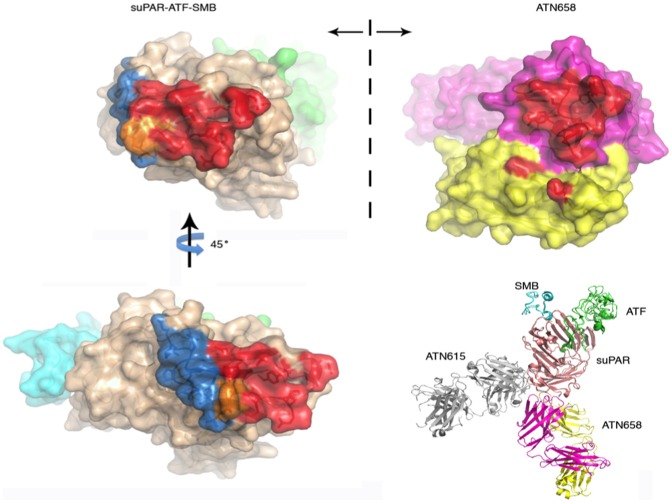
X-ray structure of ATN-658-uPAR-ATF-SMB tertiary structure. (A) The 1.6 Å structure of the ATN-658 Fab at two orthogonal views. Light chain is shown in light blue and heavy chain in dark blue. (b) Stereo view of the ATN-658 Fab in complex with suPAR (magenta) in the presence of ATF (cyan) and SMB (green) of vitronectin. All figures were made by PyMOL. (c) Interaction of uPAR–ATN-658 Fab in stereoview. Selected contacting residues in stick representation; hydrogen bonds are shown in dashed lines. (d) Open-book view of the interface between suPAR (left) and atn658 Fab (right). The Fab heavy and light chains are pink and yellow, respectively, whereas the suPAR, ATF and SMB are rose, green and cyan, respectively. The binding interface between suPAR and Fab are colored as red for atn658, blue for atn615 and orange for the overlapping epitope of these two antibodies.

Despite the limited resolution of 4.5 Å, the ATN-658-uPAR-ATF-SMB quaternary complex has high quality. The inherent glycans of uPAR at amino acid residue 52, 172, and 200 were clearly visible in the 2Fo-Fc electron density map. Three N-acetylglycosamine glycan residues were built into a large piece of electron density around the uPAR glycosylation site at residue 52. This is the largest glycan structure observed so far among all the published uPAR-ligand structures at this position. Besides the antibody, the other three components in the quaternary complex, uPAR-ATF-SMB, adopt the same conformation as previous published structures (3BT1 and 3BT2) [47] with an overall RMSD of 0.8 Å. ATF binds in the central pocket of the receptor comprised of all of three domains [33]. The SMB binds to the outer side of the DI β sheet, and also to part of the DII domain [47]. Both the ATF and SMB are located on the top of the receptor (Fig. 4), but without direct overlap between them. Crystal packing shows that ATF and SMB from one complex intertwined into the cleft formed by the ATF and SMB from another symmetry-related ATN-658-uPAR-ATF-SMB complex with hydrophilic and hydrophobic interactions. The buried area between these two complexes is quite large (3033 Å^2^). This extensive crystal contact of the SMB explains the requirement for the SMB to generate the current crystal form.

The structure of ATN-658-uPAR-ATF-SMB quaternary complex corroborates the biochemical mapping studies but also reveals several unexpected features of both the epitope and ATN-658 itself. The epitope of ATN-658 is restricted to the DIII domain at the bottom (near the C-terminus) of the receptor (Fig. 4), and consists of three segments of the uPAR DIII domain: 1) the C-terminal segment of uPAR (residues Gly270, His273, and Asp275); 2) uPAR DIII domain β strand IIIA (residues Gln193 and Tyr195); and 3) uPAR DIII domain β strand IIIA (residues Phe211, Leu212 and Asp214). Among these three segments, the C-terminal segment of uPAR has direct interaction to CDR H3 loop of ATN-658 and appears to be the most important epitope for antibody recognition. These results are consistent with the alanine scanning mutagenesis results (Fig. 2) and the alignment of non-primate of uPAR sequence (Table 3). On the ATN-658 side, both the heavy and light chains of ATN-658 are involved in antigen binding. However, the heavy chain CDR are the primary structural determinants that recognize suPAR. The light chain CDR play a minor role in the binding, recognizing only the last C-terminal residue of uPAR, Asp275. Such recognition modes relying on the heavy chain have been observed in other antibody-antigen complexes [48], [49].

### ATN-658 has structural and sequence homology to the uPAR binding region of CD11b(αM) and inhibits binding to α5β1 and RGD mediated adhesion to fibronectin

**Figure 5:**
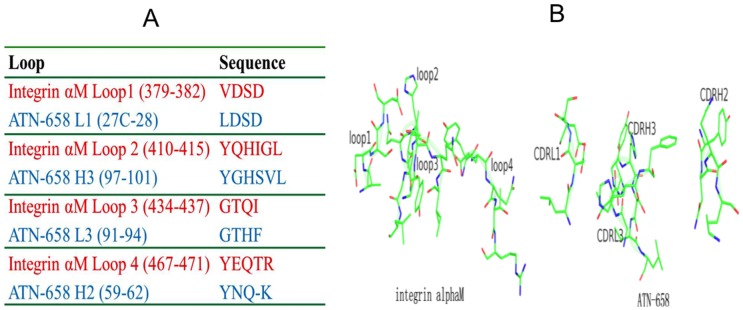
Sequence and 3-dimensional structural similarity between αM binding loops and ATN-658 CDR loops. (A) Sequence alignment of integrin αM loops to ATN-658 CDR loops. (B) Similar spatial arrangement between integrin αM loops and the ATN-658 CDR loops. Structure of αM was a homology model built from the known integrin structures (http://prosite.expasy.org/cgi-bin/pdb/get-pdb.pl?1a8x).

We found that short stretches of the ATN-658 CDR sequences (H2, H3, L1, L3) align and have a high degree of homology with the β-propeller domain of the integrin, CD11b (αM) (loops 1, 2, and 3, Fig. 5A). In addition, the spatial arrangements of these loops also superimpose well in three-dimensions, based on the current ATN-658 crystal structure and a homology structure model of αMβ2 (Fig. 5B). It would have been very difficult to detect this homology based on the alignment of linear amino acid sequences since these short stretches of amino acids assemble from the various CDR loops only in the three-dimensional structure of ATN-658 bound to suPAR. This suggests that ATN-658 may be an antagonist of the αMβ2-uPAR interaction. Further experimental verification is currently underway.

**Figure 6:**
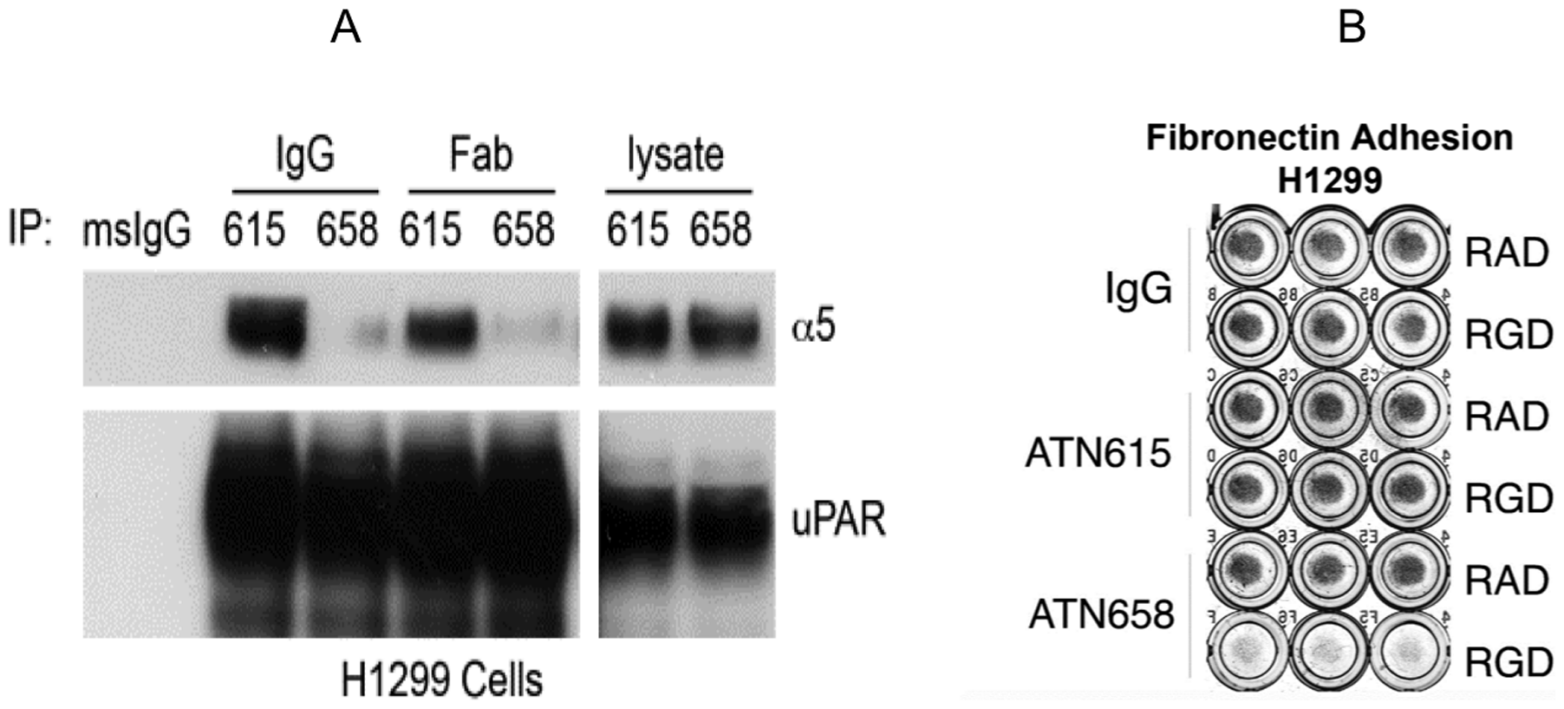
ATN-658 inhibits α5β1 mediated binding to uPAR and adhesion to fibronectin. (A) ATN-658, but not ATN-615, inhibits coIP of uPAR with α5. HT1080 cells were lysed in 1% Triton X-100 lysis buffer and the lysates were immunoprecipitated with indicated antibodies, followed by separation on SDS-PAGE and blotting for uPAR (R2) and integrin α5. Data shown are representative of three independent experiments. White lines indicate that intervening lanes have been spliced out. (B) ATN-658, but not ATN-615, interferes with H1299 uPAR-expressing cells adhesion on Fn in the presence of RGD peptide. H1299 cells pre-treated with RGD or RAD peptides (500 µM) were seeded onto Fn-coated 96-well plates together with antibodies ATN-615 or ATN-658. After incubation for 1 hr at 37°C, plates with triplicate determinations were washed, and attached cells were fixed and stained with Giemsa. All the above experiments were performed at least three times with similar results.

We also found that ATN-658 blocks the interaction of uPAR with another integrin, α5β1. ATN-658 and a control anti-uPAR antibody, ATN-615, both in IgG and Fab forms, were used to immunoprecipitate (IP) uPAR from cell lysates of H1299, a human non-small lung carcinoma cell line that expresses high levels of uPAR. The resulting immunoprecipitates were analyzed by Western blot for uPAR and α5 integrin subunits. ATN-615 was able to IP both uPAR and α5 integrin (Fig. 6A). However, ATN-658 was only able to IP uPAR, but not α5, and thus appears to block the interaction of uPAR with α5 integrin. To validate this result, a fibronectin adhesion assay was carried out. Adhesion of H1299 cells to fibronectin is mediated to a large extent by the α5β1 integrin and this adhesion can be completely blocked by an α5β1-blocking antibody (5H10-27) [29], [50]. This α5β1-mediated fibronectin adhesion can also be abrogated by an integrin binding RGD peptide. However, we observed that uPAR over-expression in H1299 cells can antagonize the effect of the RGD peptide, and increase cell adhesion to fibronectin in the presence of RGD-containing peptides [50]. Based on this uPAR-enhanced adhesion and other observations, uPAR was proposed to interact with the bent conformation of integrin α5β1 and render it capable of engaging fibronectin adhesion [29]. This effect of uPAR-enhanced adhesion is completely inhibited by ATN-658, but not by control IgG or ATN-615 (Fig. 6B). This suggests that ATN-658 can also block the uPAR-α5β1 interaction which was unexpected since the region of uPAR that has been implicated in the α5β1 interaction resides between aa 240–248 [15] whereas ATN-658 binds to aa 268–275. However, previous studies using a peptide derived from αM (M25) that inhibited the uPAR-αM interaction [51] demonstrated that inhibiting the uPAR-αM interaction on leukocytes also blocked the uPAR-α5β1 interaction, which suggests that ATN-658 may broadly impact uPAR-integrin interactions.

## Discussion

In this study, we have identified an epitope in uPAR with a previously unrecognized function that serves as the binding site for the novel therapeutic uPAR antibody, ATN-658. Over the past several decades, several uPAR antibodies have been identified that bind to human uPAR with high affinity. However, ATN-658 is the first uPAR antibody to demonstrate consistent robust antitumor effects across a variety of tumor models [22], [26]–[28] that include not only inhibition of invasion and metastasis but also inhibition of proliferation and induction of apoptosis. The epitope for ATN-658, aa 268–277 of uPAR, resides in DIII and contains a small 6-mer disulfide loop near the glycolipid anchor of uPAR. Another anti-uPAR antibody, ATN-615, also binds to the DIII domain of uPAR [34], but does not show any antitumor effects *in vitro* or *in vivo*. This non-inhibitory antibody recognizes a different non-overlapping set of epitope residues compared to ATN-658 and emphasizes the importance of targeting the correct uPAR epitope for cancer therapy. We have also evaluated other uPAR antibodies that block uPA binding to uPAR (e.g. ATN-617) in various tumor models and these also have poor antitumor effects *in vivo*. Based on these observations, ATN-658 has now been humanized (huATN-658) and a first-in-man study is being planned for the near future.

Of broader significance is the mechanism of how ATN-658 exerts its antitumor effects at the molecular level. The observation that the interaction of ATN-658 with uPAR closely mimics that of CD11b (αM; Mac-1; CR3) with uPAR has broad implications for the role of uPAR in tumor progression. αM forms a heterodimer with β2 (αMβ2) and is expressed on the surface of many leukocytes involved in the innate immune system [52], [53]. αMβ2 mediates inflammation by regulating leukocyte adhesion and migration [52], [53]. Recently, CD11b expression has been demonstrated to define a subpopulation of bone marrow derived myeloid cells (BMDC) that seed the pre-metastatic niche and without which tumor metastasis is unable to progress [54]. The CD11b positive BMDC may actually encompass several subpopulations of cells, some of which act as suppressors to dampen cytotoxic T cell response and therefore allow tumors to progress [55]. In addition, CD11b-positive cells may secrete factors that drive tumor progression and subpopulations have also been demonstrated to be involved in tumor resistance to anti-angiogenic therapy [56].

Historically, uPAR has been implicated in the metastatic process and conceptually, this implication has focused on its role in invasion and migration prior to intravasation or during the process of extravasation but not once cells have extravasated and seeded a metastatic site. The fact that ATN-658 mimics the CD11b-uPAR interaction suggests that ATN-658 may block this interaction and suggests several hypotheses as to how uPAR may actually function to promote metastasis. When treating a patient with advanced cancer, it needs to be assumed that metastases are already present when treatment is started even if they are not detected radiographically. Thus, inhibiting the progression of established metastases may be more therapeutically relevant than trying to interfere with the metastatic process. If the uPAR-CD11b interaction is central to metastatic outgrowth, ATN-658 may be targeting the most clinically relevant and actionable aspect of metastasis.

This study leads to several hypotheses of how uPAR-CD11b may promote metastasis. One hypothesis is that uPAR on a tumor cell may interact with CD11b on a leukocyte in the pre-metastatic niche in *trans* and this interaction may mediate survival and outgrowth of the uPAR expressing tumor cells, possibly by dampening T cell or innate immune response at the metastatic site. An alternative hypothesis is that CD11b is expressed directly on a tumor cell so that uPAR and CD11b interact in *cis*. The contribution of each of these interactions to tumor progression and the molecular mechanisms that are downstream of uPAR-CD11b binding remain to be elucidated and are a major focus for future studies.

**Figure 7:**
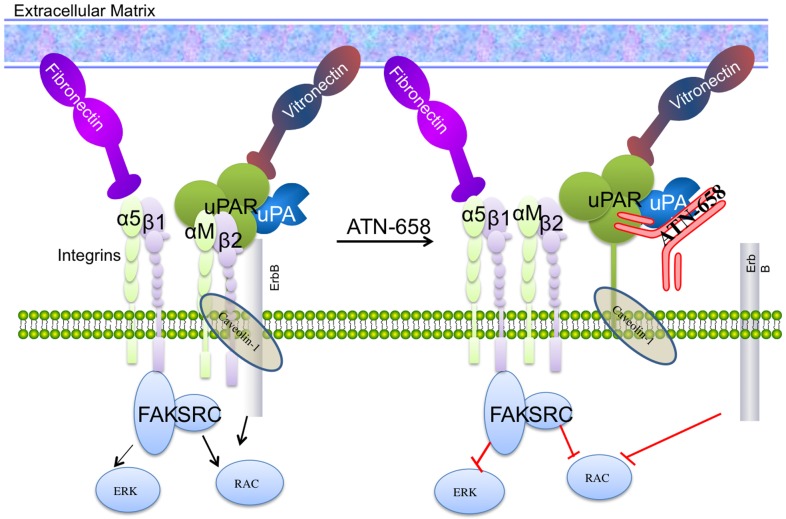
Schematic of ATN-658 disruption of uPAR signalosome.

In our study, we also observe the ability of ATN-658 to inhibit the interaction of uPAR with α5β_1_ in H1299 tumor cells as well as antagonize RGD mediated anti-adhesive effects of tumor cells interacting with fibronectin (Fig. 6). Although we cannot rule out a steric effect of ATN-658 on the uPAR interaction with a5β1, we hypothesize that ATN-658 may alter global integrin clustering and signaling through the inhibition of the CD11b-uPAR interaction. In that regard, a peptide, M25, that blocked the uPAR-CD11b interaction was demonstrated to also inhibit the interaction of uPAR with β1 integrins (32). In addition, we also demonstrate that ATN-658 blocks CD11b mediated adhesion of U937 cells. Our previous studies using both unbiased and biased approaches demonstrated that ATN-658 inhibits integrin signaling *in vitro* and *in vivo* as well as co-localization of α5β1 and uPAR [22], [27]. Our data suggest that the effects of ATN-658 on perturbing the uPAR-α5β1 interaction is indirect and direct binding of uPAR to integrin α5β1 or any other integrin using purified components has not been demonstrated [57], suggesting that these interactions require multiple components present at the cell surface. The existence of a signalosome containing uPAR has been postulated by D'Allessio and Blasi [58] and the data obtained to date supports the hypothesis that ATN-658 disrupts the signalosome leading to global effects on integrin signaling and inhibition of tumor progression (Fig. 7). uPAR may behave as a scaffolding protein in the assembly of this signalosome. Although the exact temporal order and sets of interactions that mediate these effects remain to be elucidated, these observations strongly support uPAR as a global mediator of integrin signaling and interactions as well as a cancer target that will affect multiple tumor progression pathway [22], [26]–[28].
