# Correction: Identification of a New Epitope in uPAR as a Target for the Cancer Therapeutic Monoclonal Antibody ATN-658, a Structural Homolog of the uPAR Binding Integrin CD11b (αM)

**DOI:** 10.1371/journal.pone.0098490

**Published:** 2014-05-19

**Authors:** The PLOS ONE Staff

The second author’s name is incorrect. The correct name is “Cai Yuan.” The correct citation is: Xu X, Yuan C, Wei Y, Donate F, Juarez J, et al. (2014) Identification of a New Epitope in uPAR as a Target for the Cancer Therapeutic Monoclonal Antibody ATN-658, a Structural Homolog of the uPAR Binding Integrin CD11b (αM). PLoS ONE 9(1): e85349. doi:10.1371/journal.pone.0085349

Figure 4 is incorrect. The authors have provided the correct version below.

**Figure 4:**
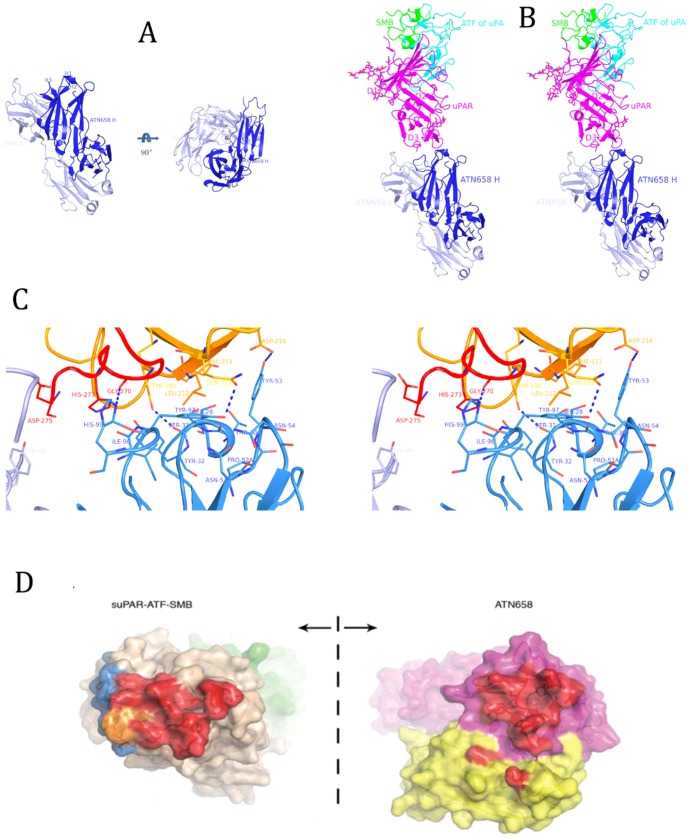
X-ray structure of ATN-658-uPAR-ATF-SMB tertiary structure. (A) The 1.6 Å structure of the ATN-658 Fab at two orthogonal views. Light chain is shown in light blue and heavy chain in dark blue. (b) Stereo view of the ATN-658 Fab in complex with suPAR (magenta) in the presence of ATF (cyan) and SMB (green) of vitronectin. All figures were made by PyMOL. (c) Interaction of uPAR–ATN-658 Fab in stereoview. Selected contacting residues in stick representation; hydrogen bonds are shown in dashed lines. (d) Open-book view of the interface between suPAR (left) and atn658 Fab (right). The Fab heavy and light chains are pink and yellow, respectively, whereas the suPAR, ATF and SMB are rose, green and cyan, respectively. The binding interface between suPAR and Fab are colored as red for atn658, blue for atn615 and orange for the overlapping epitope of these two antibodies.
