# Correction: Identification of a New Epitope in uPAR as a Target for the Cancer Therapeutic Monoclonal Antibody ATN-658, a Structural Homolog of the uPAR Binding Integrin CD11b (αM)

**DOI:** 10.1371/journal.pone.0099911

**Published:** 2014-06-04

**Authors:** The PLOS ONE Staff

There are 2 errors in Table 1. The following PDB codes are missing: 4K23 for ATN-658 Fab and 4K24 for ATN-658-uPAR-ATF-SMB. Please see below for a corrected version.

**Table 1:** Statistics of X-ray data collection and structural model refinement.

Crystals	ATN 658 Fab	ATN-658-uPAR-ATF-SMB
**X-ray source**	APS SER-CAT beamline 22-ID	**BNL x29**
**Temperature (K)**	100	**100**
**Resolution range**	31.45-1.60 (1.64-1.60)^a^	**26.90- 4.50 (4.66-4.50)** a
**Wavelength (Å)**	1.04	**1.04**
**Space group**	P12_1_1	**H32**
**Cell parameters (Å)**	37.31, 132.30, 46.83	**164.65, 164.659, 391.582**
**Unique reflections**	49312 (4021)	**12451 (1221)**
**Rmerge (%)^b_^**	0.094 (0.290)	**0.139 (0.841)**
**Completeness (%)**	85.7(70.0)	**99.7(100.0)**
**Average I/σ**	9.2 (4.65)	**16.14 (2.03)**
**Data redundancy**	3.0 (2.6)	**4.4 (4.5)**
**Model refinement:**		
**R-factor/Rfree (%)**	19.8/24.6	**20.7/29.3**
**Overall B-factors (Å^2^)**	23.0	**61.436**
**Protein B-factors (Å^2^)**	21.3	**259.694**
**Solvent B-factors (Å^2^)**	39.4	
**R.m.s.d. from ideal bond length (Å)**	0.012	**0.013**
**R.m.s.d from ideal bond angles (°)**	1.407	**2.018**
**Ramachandran plot, % residues in regions:**		
**favored**	93.8	**84.5**
**allowed**	5.7	**13.7**
**outlier**	0.5	**1.8**
**PDB ID**	4K23	**4K24**

a Numbers in the parentheses are for the highest resolution shells; ^b^ R_merge_ = ΣhΣi|Ii(h)-<I(h)>|/ΣhΣiIi(h), where <I(h)> is the mean intensity of reflection h.
